# The Bitemporal Lens Model—toward a holistic approach to chronic disease prevention with digital biomarkers

**DOI:** 10.1093/jamiaopen/ooae027

**Published:** 2024-04-08

**Authors:** Filipe Barata, Jinjoo Shim, Fan Wu, Patrick Langer, Elgar Fleisch

**Affiliations:** Centre for Digital Health Interventions, ETH Zurich, Zürich, Zürich, 8092, Switzerland; Centre for Digital Health Interventions, ETH Zurich, Zürich, Zürich, 8092, Switzerland; Centre for Digital Health Interventions, ETH Zurich, Zürich, Zürich, 8092, Switzerland; Centre for Digital Health Interventions, ETH Zurich, Zürich, Zürich, 8092, Switzerland; Centre for Digital Health Interventions, ETH Zurich, Zürich, Zürich, 8092, Switzerland; Centre for Digital Health Interventions, University of St. Gallen, St. Gallen, St. Gallen, 9000, Switzerland

**Keywords:** preventive medicine, digital biomarkers, digital health, wearable technology, mobile health, health promotion, predictive analytics, health monitoring, personalized prevention, precision health, biomedical sensors, telehealth, digital health interventions, cardiovascular disease

## Abstract

**Objectives:**

We introduce the Bitemporal Lens Model, a comprehensive methodology for chronic disease prevention using digital biomarkers.

**Materials and Methods:**

The Bitemporal Lens Model integrates the change-point model, focusing on critical disease-specific parameters, and the recurrent-pattern model, emphasizing lifestyle and behavioral patterns, for early risk identification.

**Results:**

By incorporating both the change-point and recurrent-pattern models, the Bitemporal Lens Model offers a comprehensive approach to preventive healthcare, enabling a more nuanced understanding of individual health trajectories, demonstrated through its application in cardiovascular disease prevention.

**Discussion:**

We explore the benefits of the Bitemporal Lens Model, highlighting its capacity for personalized risk assessment through the integration of two distinct lenses. We also acknowledge challenges associated with handling intricate data across dual temporal dimensions, maintaining data integrity, and addressing ethical concerns pertaining to privacy and data protection.

**Conclusion:**

The Bitemporal Lens Model presents a novel approach to enhancing preventive healthcare effectiveness.

## Introduction

Already in the 16th century, Santorio Santorio, a physician from Venice and the University of Padua identified that self-measurement is a critical component of health empowerment, guiding personal health interventions.^1^ Santorio famously dedicated his efforts to monitoring his body and tracking changes in weight resulting from food intake and excretion. Through emphasizing the importance of weight monitoring, Santorio linked body measurement to personal well-being by demonstrating how it could help maintain a consistent body weight.^1^ Today, it is more important than ever to empower individuals to take charge of their health in order to realize the full potential of disease prevention strategies. Chronic diseases, which are often caused by lifestyle choices such as chronic obstructive pulmonary disease, type 2 diabetes, and cardiovascular disease (CVD), account for most global deaths, morbidity, and health-related financial burdens.^2^ Therefore, there is a growing emphasis on prioritizing disease prevention,^3^ and the need for daily management strategies.

With the widespread use of mobile technologies, such as wearable sensors, mobile apps, social media, and location-tracking tools, new opportunities for health self-tracking have emerged.^4^ Similar to the introduction of the weight scale, the data collected by consumer-centric devices enable patients and healthy individuals alike to form their judgments about their health and thus become empowered and active individuals.^5^ In particular, objective, quantifiable physiological and behavioral data (eg, physical activity, heart rate, electrodermal activity) that are collected and measured by means of digital devices,^4^ so-called *digital biomarkers*, promise continuous, noninvasive health monitoring at a much lower cost than traditional episodic follow-ups and clinic visits. This more proactive approach to health management provides individuals with real-time data on their health status, thereby addressing the potential to meet the health monitoring needs of the at-risk and aging population worldwide.

**Figure 1.**
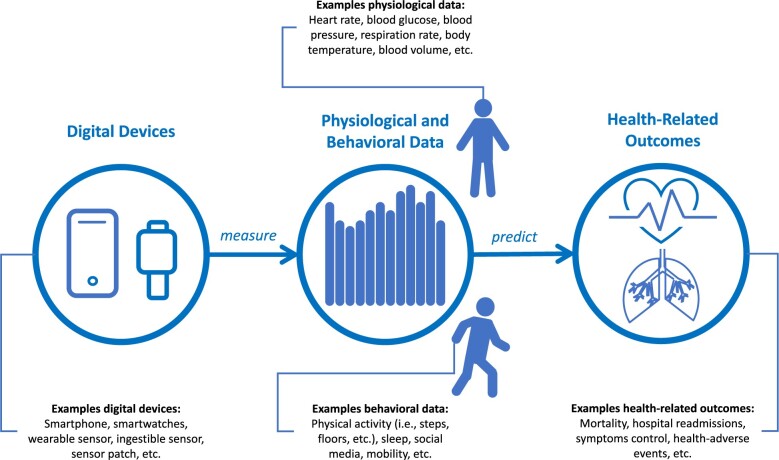
The anatomy of digital biomarkers.

Whereas digital biomarkers go by many definitions,^6^ they can generally be understood as a conglomerate of 3 components, (1) a digital device, which (2) collects physiological and/or behavioral data that (3) can predict, explain, or infer a health-related outcome (cf. Figure 1). From a technology point of view, digital biomarkers require specific software and hardware, algorithms, databases, and user communication. In most cases, developing digital biomarkers involves collecting longitudinal unstructured data from multiple patients with existing conditions and carrying digital devices. To be of use to physicians and patients, raw sensor data collected by these devices must be processed and aggregated. This processed data can then be associated with or utilized to predict health-related outcomes, often using machine learning. Machine learning algorithms are indispensable for deriving meaningful insights from vast amounts of data, including longitudinal data collected during digital biomarker research. These algorithms can reveal hidden patterns in the data, leading to a deeper understanding of an individual’s health status and facilitating more accurate medical assessment. After their training phase, these algorithms can be integrated into applications, either directly on devices or through server-side deployments.

Effective prevention necessitates the recognition of the need for change. This awareness is pivotal in initiating preventive measures or interventions, which can be implemented using digital biomarkers.

In fact, many digital biomarkers are used in the realm of tertiary prevention, where the focus lies on reducing the effects of disease once established in an individual.^7^ In this context, digital biomarkers help devise monitoring applications,^8–11^ functional assessments,^12–14^ and thus, give rise to preventive interventions.^15–17^ In patients with diabetes, for instance, digital biomarkers can monitor blood glucose levels and detect hypoglycemia or hyperglycemia early on, enabling prompt intervention such as medication adjustments or dietary changes to prevent serious complications.^18^^,^^19^ Digital biomarkers, however, are also employed in the realm of secondary prevention,^20–22^ which involves the early detection of diseases.^7^ In this case, digital biomarkers that track physical activity, sleep patterns, and heart rate can detect early indicators of mental health conditions like depression, leading to early intervention and prevention of the condition from worsening.^23^ Finally, digital biomarkers have also been employed in the realm of primary prevention using large population-based data,^24–26^ which is aimed at preventing the onset of diseases.^7^ For instance in primary prevention, wearable-device-measured physical activity profiles are associated with systemic inflammation, biological age, and all-cause mortality.^26^

Most digital biomarkers are, however, currently designed primarily for monitoring or predicting a single health outcome, facilitating interventions targeted at specific clinical results. Consequently, the majority of evaluated digital biomarker-based interventions today predominantly focus on enhancing either immediate, critical clinical outcomes like cardiac function or on those factors impacting long-term well-being such as physical activity.^17^ Concurrently, health can deteriorate rapidly (eg, infections) or gradually (eg, progressive decline in bodily functions) over extended periods. This necessitates interventions of varying urgency tailored to address both immediate and long-term health outcomes. This article posits that a comprehensive approach to digital biomarker-based prevention must encompass both temporal aspects and integrate the analysis of the interplay between proximal and distal health outcomes. To this end, we first revisit the prevalent approach in developing digital biomarkers for prevention. Secondly, we introduce the complementary recurrent-pattern model. Finally, we discuss how both models can be integrated into one approach for developing digital biomarkers for prevention—the Bitemporal Lens Model.

## The oversight of preventative measures in healthcare

While digital biomarkers show promise in empowering individuals to take control of their health, most of the current developments have primarily focused on disease management rather than prevention.^27^ Additionally, research into methods of using digital biomarkers for preventive purposes is still in its infancy.^28–30^ This emphasis on disease management over prevention is historically rooted, with the healthcare industry and medical research traditionally being more inclined towards the treatment of diseases as opposed to their prevention.^31^ For example, in 2015, the United States allocated only 3% of its total healthcare expenditures to preventive services.^32^ Also, prevention demands long-term studies and extensive data for proving efficacy, as it involves tracking health over time to demonstrate disease prevention, making it more difficult (eg, lack of adherence) and costly than disease management studies.^33^ Further challenges in digital biomarker development for prevention may encompass difficulty in developing highly sensitive digital biomarkers,^29^ especially with application to pre-disease states. Also, the relatively nascent understanding and interpretation of indicators for pre-disease risks as compared to established disease biomarkers may further complicate the development of digital biomarkers for prevention.

In addition to the obvious strategy of directing more investment towards prevention, a promising strategy to surmount these challenges could so often involve the application of artificial intelligence and machine learning techniques. Machine learning-based approaches have not only shown in the last decade their tremendous potential in the field of radiology but have also demonstrated efficacy in various tasks using digital biomarkers, eg, in augmenting the detection capabilities of digital sensors.^34^ Despite the challenges associated with conducting longitudinal studies, contemporary efforts in establishing biobanks could benefit from integrating data from devices owned by participants, such as wearables or smartphones. Such an approach is cost-efficient and poses limited additional burdens and risks for participants. A pertinent example of this is the “All of Us” research program,^35^ which notably includes the longitudinal collection of smartwatch data. In conclusion, the formulation of specialized solutions to these challenges necessitates the extension and enrichment of the prevailing model and knowledge regarding the application of digital biomarkers for prevention, a domain where our work provides novel contributions.

## The conventional change-point model

The prevalent approach in developing digital biomarkers for prevention follows a paradigm where changes in biomarker values must occur to signal a decline in health status and trigger an intervention.^8^^,^^19^^,^^36^ This change in values can be defined by a specific threshold of biomarker values (also known as change point^37^) at which the medication or treatment must be introduced or adjusted, eg, C-reactive protein levels >10 mg/L are indicative of an acute infection or inflammation.^38^ To facilitate the discussion in this work, we refer to this paradigm as the “change-point model.”

The change-point model suffers from several limitations. First, it assumes that when a critical change is identified there is enough time to intervene and prevent or mitigate a health-adverse outcome. The second assumption is that there is no lag time between the levels of the observed surrogate digital biomarkers and the actual health status. If this assumption does not hold, it means that the critical values of the surrogate digital biomarkers may only become apparent after the critical health state has already been reached, which could limit the effectiveness of interventions. Third, the efficacy of digital biomarkers is evaluated against pre-established gold standard biomarkers. Their actual effectiveness, however, cannot be accurately assessed as they are inherently limited by the effectiveness or ineffectiveness of the gold standard, respectively.

## Recurrent behaviors and recurrent patterns in time-series data

Twin studies have provided evidence that genetic differences contribute to approximately 25% of the variation in human adult lifespan, underscoring the significance of lifestyle choices and recurrent behaviors in determining longevity.^39^ Recurrent behaviors play a substantial role in our well-being, where healthy habits such as regular sleep,^40^ regular exercise,^41^ and a balanced diet^42^ promote overall health, while unhealthy behaviors like inactivity^43^ and substance abuse can lead to various health issues.^44^ For instance, circadian rhythms, also known as the “body’s clock,” are regular patterns of biological activity that naturally occur within the body and follow a 24-hour cycle.^45^^,^^46^ Our behavior and lifestyle choices (eg, shift work^47^) can lead to repeated disruptions in circadian rhythms, which are linked to dysregulation of immune responses and inflammation,^48^ resulting in an increased risk of developing chronic diseases.^49^ Other examples of recurrent patterns in health data include walking speed^50^^,^^51^ as well as seasonal variations in health conditions like allergies^52^^,^^53^ and certain infectious diseases.^54^ Recurring patterns can be captured through time-series data generated by the daily use of consumer-centric devices such as smartphones^55^ or smartwatches.^56^ For example, modalities like actigraphy and core body temperature measurement can be used to monitor the circadian rhythm.^57^ Identifying these recurrent patterns in time-series data can reveal well-defined processes that can be further analyzed and serve as a reliable feature for predicting future occurrences of the observed pattern.^24^ Indeed, recent studies have highlighted the promise of examining recurrent patterns using time-series data of digital biomarkers, offering insights into health risks and longevity.^40^^,^^58–60^ For example, Windred et al demonstrated that actigraphy-derived sleep regularity is a stronger predictor of mortality risk than sleep duration.^40^

On this basis, we argue that these repetitive patterns that have such a profound impact on our health and lifespan can be detected using data captured by digital biomarkers. This gives rise to the recurrent-pattern model, a different paradigm from which digital biomarkers can promote prevention. Rather than looking for when values are indicative of a change in health deterioration, this paradigm puts forward the investigation of recurrent patterns in digital biomarker data that anticipate and can be associated with those changes. This approach brings several advantages.

First, the recurrent-pattern model focuses on the idea of prevention, ie, recognizing recurrent patterns in digital biomarker data that negatively affect our health before actual deterioration occurs. Second, this enables the study of associations in recurrent patterns in digital biomarker time series data with repetitive behaviors, elucidating their connection with adverse health outcomes. Third, such an approach enables personalized interventions targeted at unhealthy behaviors instead of raised values. This reduces the time pressure associated with intervention, such as the time to treatment and the time to onset of deterioration.

Whereas the recurrent-pattern model may offer some advantages over the change-point model, it is important to acknowledge its limitations and underlying assumptions. Firstly, it does not give priority to monitoring sudden changes, which limits its ability to detect significant changes that are crucial for preventing acute health problems and acting with urgency. Secondly, the reliability of identifying recurrent patterns in the data, as assumed by the recurrent-pattern model, can be compromised by issues like noise or insufficient sensor sensitivity, making consistent detection challenging. Thirdly, it assumes that all trajectories of chronic diseases are influenced by oscillations caused by recurrent behaviors, which may not hold for certain illnesses. Lastly, generalizing recurrent patterns in the context of digital biomarkers can be challenging due to the significant heterogeneity observed in individuals and the complexity of diseases.

## The Bitemporal Lens Model and future recommendations

**Figure 2.**
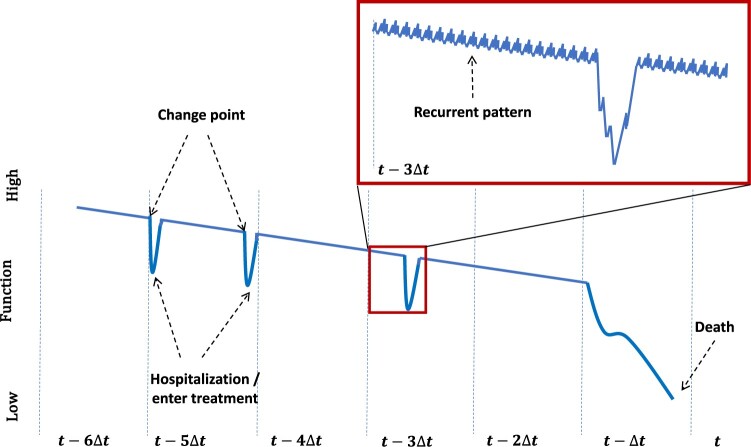
The Bitemporal Lens Model applied to the trajectory of chronic disease. Three different health deterioration episodes, before death occurs. The start of these episodes is characterized by a change point. A detailed view uncovers the recurrent patterns in the data preceding the health deterioration episode.

On this basis, we argue that both the change-point and recurrent-pattern models are crucial for prevention, as they offer distinct perspectives on the timing of preventive measures. Thus, to the best of the authors’ knowledge, we propose a novel and comprehensive method for health prevention that combines the previously mentioned two models, termed the Bitemporal Lens Model. Figure 2 shows an exemplary manifestation of the Bitemporal Lens Model in the trajectory of chronic disease as introduced by Murray et al.^61^

The Bitemporal Lens Model not only integrates the attributes of established models but also fosters novel opportunities for individualized customization. To elucidate the inherent functionalities of the Bitemporal Lens Model, we consider the following illustrative scenario: We aim to longitudinally monitor a cohort deemed healthy yet predisposed to CVD, eg, characterized by prehypertension stage of systolic and diastolic blood pressure readings within 120-139/81-89 mm Hg and an overweight body mass index between 25-29.9 kg/m^2^. Within this group, some individuals may eventually develop CVD, potentially leading to serious health complications such as myocardial infarction, heart failure, and arrhythmias. Employing the change-point lens aspect of the model, we focus on tracking critical disease-specific parameters like increases in blood pressure or changes in heart rate variability, to identify early signs of CVD or related health-adverse events indicating the potential need for urgent interventions. Concurrently, the recurrent pattern lens aspect concentrates on observing lifestyle and recurrent behavioral patterns, such as circadian disruption, physical activity, and alcohol consumption, to identify risk factors frequently preceding CVD onset and related complications. This lens enables targeted behavioral interventions. The integration of these 2 aspects, that is the Bitemporal Lens Model, gives rise to an additional dimension to the individual lenses by enabling a personalized risk assessment for each subject in the cohort. By considering both the current status of disease-critical parameters (such as blood pressure and heart rate variability) and historical patterns of behavior (such as physical activity and alcohol consumption) the Bitemporal Lens Model allows for an individualized retrospective analysis and future prediction of health trajectories. Thus, the bitemporal analysis can shed light on how CVD develops in at-risk populations by understanding individual health trajectories contributing to the broader understanding of disease dynamics. As a result, the Bitemporal Lens Model enables the development of personalized intervention plans based on individual risk profiles. The Bitemporal Lens Model may be visualized using 2 parallel timelines: the “First Temporal Lens” for the current perspective, marked with data points for recent observations on critical disease-specific parameters, and the “Second Temporal Lens” for the historical perspective, charted with data points and annotations for past events and trends of disease-relevant lifestyle and recurrent behaviors. This dual-timeline approach provides a comprehensive view, combining present developments with historical data to enhance understanding of trends, changes, and future predictions.

The Bitemporal Lens Model, while offering in-depth insights through dual-temporal data analysis, faces several challenges. These include managing the complexity and volume of data from 2 time frames, ensuring data quality and consistency, and integrating diverse data sets. A critical aspect is the sophisticated synchronization of disparate data sets, aligning real-time physiological parameters with longitudinal behavioral data. For change-point models, data collection should capture the transition to health deterioration, requiring careful subject recruitment and handling of imbalanced data. Recurrent-pattern models focus on identifying stable patterns and associating them with adverse health outcomes, necessitating longitudinal data collection and addressing issues of participant adherence and missing data. Moreover, the Bitemporal Lens Model introduces extra software and infrastructural needs due to the importance of frequently updating data sets, eg, maintenance, storage, and ensuring data congruence and integrity. Over-reliance on historical data in models could lead to incorrect predictions about future trends. Finally, ethical considerations and the safeguarding of privacy, particularly in the handling of sensitive health-related behavioral and physiological data, are of utmost importance for its implementation, including accuracy to uphold the model's predictive validity and reliability in a healthcare setting.

## Conclusions

Despite the potential of digital biomarkers, current applications are predominantly unidimensional, focusing on singular health outcomes that guide interventions toward clinical endpoints. This narrow focus yields interventions that are either aimed at critical, short-term clinical outcomes, such as optimizing cardiac function or at improving long-term health determinants, such as encouraging regular physical activity. In this work, we propose a novel bitemporal approach to digital biomarkers within preventive healthcare. Such an approach would not only accommodate the immediate clinical necessities but also encompass a long-term perspective, thereby enabling an individual analysis of risk profiles and health trajectories.

## Data Availability

No new data were generated or analyzed in support of this research.
